# 891. Work-related Quality of Life in Burn Center Staff: Variations & Associations Across a Year

**DOI:** 10.1093/jbcr/irag033.377

**Published:** 2026-04-06

**Authors:** Emily Beutler, Micayla Kotarski, Callie M Thompson

**Affiliations:** University of Utah, Salt Lake City, UT; University of Utah Health Burn Center, Salt Lake City, UT; University of Utah Health, Salt Lake City, UT

## Abstract

**Introduction:**

Achieving optimal outcomes in burn care requires a specialized interdisciplinary team. In recent years, concerns for the short- & long-term stability of the burn workforce have been rising as burnout in the entire healthcare industry has reached all-time highs. To understand our burn center Work-Related Quality of Life (WRQoL) & the factors that may impact it, we initiated a year-long quarterly survey of our ABA-Verified Regional Burn Center staff. Here, we describe the novel findings of that survey series.

**Methods:**

After IRB approval, we sent an anonymous quarterly email survey to all burn center staff at our ABA-verified Regional Burn Center between May 2024 and June 2025. We collected minimal demographic data & inquired about WRQoL, feelings of safety, & intention to leave. Standard statistical analysis was performed with chi-squared analysis used to compare categorical variables.

**Results:**

Response rates varied from 25-41%. Demographics of respondents are in Table 1. The responses to the WRQoL question regarding overall satisfaction with working life did not significantly change over time with 39-59% reporting dissatisfaction, strongly or otherwise. Stable percentages reported feeling excessive stress (38-53%) and consider leaving their job at least every few months (55-57%) A verbal assault at work in the last quarter was reported by 29-47% and such an assault was associated with reporting less overall satisfaction, 29 vs. 66% (*p*<.001) and thinking about leaving the job at least every few months, 76 vs. 45% (*p*<.001).

**Conclusions:**

Results from our burn center indicate consistent and concerning levels of stress and dissatisfaction among our interdisciplinary staff. It is particularly concerning that a majority think about leaving the burn center quite regularly. It is also worrisome that so many have reported verbal assaults in the workplace and that these events are associated with frequent thoughts of leaving the job and dissatisfaction with their working life. While we are unable to draw causation from this work, this particular finding is worthy of additional intervention with increased support for those who experience these events to evaluate whether these associations can be mitigated.

**Applicability of Research to Practice:**

Understanding the WRQoL in burn center staff is critical to future efforts to ensure the stability of this expert workforce.

**Funding for the study:**

N/A.

